# Monocytic Cell Variations in Postallotransplant Environment: Balancing Graft Versus Host Disease and Graft‐Versus‐Leukemia Effect

**DOI:** 10.1155/jimr/5303312

**Published:** 2026-09-09

**Authors:** Magdalena Karasek, Kinga Chmielewska, Anna Czyż

**Affiliations:** ^1^ Department of Hematology, Cell Therapies and Internal Medicine, Wroclaw Medical University, Wroclaw, Poland, umed.wroc.pl; ^2^ Department of Hematology, Collegium Medicum in Bydgoszcz, Nicolaus Copernicus University in Torun, Bydgoszcz, Poland, umk.pl; ^3^ Laboratory of Hematological and Transplant Diagnostics, University Hospital of Wroclaw, Wroclaw, Poland, umed.wroc.pl

## Abstract

Allogeneic hematopoietic stem cell transplantation (alloHSCT) is a potentially curative treatment for various hematologic malignancies. However, its success is critically dependent on maintaining the delicate balance between the beneficial graft‐versus‐leukemia (GvL) effect and the harmful graft‐versus‐host disease (GvHD). This review highlights the increasingly recognized role of monocyte‐derived cell populations in modulating posttransplant immune responses. We explore the functions of distinct monocytic subpopulations—classical, intermediate, and nonclassical monocytes—and their involvement in both immune reconstitution and the pathogenesis of GvHD. Intermediate and nonclassical monocytes have been associated with proinflammatory responses, particularly through induction of Th17 and Th1 cells, thus contributing to acute and chronic GvHD (cGvHD) development. In contrast, early recovery of classical monocytes correlates with improved overall survival and reduced relapse risk. Additionally, the review discusses the dual role of macrophages, with M1 macrophages enhancing inflammatory responses and GvL activity, while M2 macrophages promote tissue repair and fibrosis, particularly in cGvHD. Leukemia‐associated macrophages (LAMs) are implicated in immune escape and disease relapse due to their immunosuppressive phenotype and checkpoint expression. Finally, we examine the suppressive functions of myeloid‐derived suppressor cells (MDSCs), especially monocytic MDSCs (M‐MDSCs) and polymorphonuclear MDSCs (PMN‐MDSCs) subsets. These cells modulate T‐cell responses and may help mitigate GvHD without compromising GvL, although their exact role remains debated. Understanding the nuanced roles of these monocytic populations may open new avenues for improving alloHSCT outcomes by selectively enhancing GvL while preventing or attenuating GvHD.

## 1. Introduction

Allogeneic hematopoietic stem cell transplantation (alloHSCT) remains one of the most effective therapeutic methods with curative potential in hematologic malignancies such as acute myeloid leukemia (AML) and myelodysplastic neoplasms (MDSs) [1, 2]. Nevertheless, both relapses of malignancy and transplant‐related mortality are predominantly responsible for transplant failure [3]. The risk of relapse depends mainly on the biology of the neoplastic disease. Still, it is also modulated by the graft‐versus‐leukemia (GvL) effect on one hand and the phenomenon of tumor escape from immune surveillance on the other hand [4]. The GvL effect of recognizing and eliminating residual tumor cells by allogeneic T cells in the graft is a prerequisite for therapeutic success after alloHSCT, and loss of this effect contributes to relapse of the primary disease. In turn, transplant‐related mortality is closely associated with graft‐versus‐host disease (GvHD), the result of enhanced activity of the donor’s immune cells [4]. Thus, GvHD, alongside relapse of underlying diseases and infections, remains one of the primary causes of morbidity and mortality within 200 days following allotransplantation [5, 6]. Eventually, immunosuppressive therapy of both acute GvHD (aGvHD), diagnosed usually within the first 100 days after alloHSCT, or chronic GvHD (cGvHD) in parallel impairs the GvL phenomenon, thereby hindering the prevention or treatment of disease relapse [7].

It is worth mentioning that expanding eligibility for alloHSCT to older patients, including those over 70 years old or with comorbidities, requires the use of reduced‐intensity or nonmyeloablative conditioning protocols, which are associated with a higher risk of relapse [8]. Therefore, maintaining the GvL effect through optimized and tailored GvHD prophylaxis is of particular importance in these settings [9].

Furthermore, the source of stem cells used in alloHSCT has evolved substantially in recent years, with increasing use of haploidentical related donors facilitated by the introduction of posttransplant cyclophosphamide (PTCy)–based GvHD prophylaxis [10]. PTCy has significantly improved the safety of haploidentical transplantation by reducing both aGvHD and cGvHD, thereby broadening donor availability and transplant accessibility [11]. However, several studies have demonstrated that peripheral blood stem cell grafts, compared with bone marrow grafts, may be associated with a higher incidence of cGvHD in the setting of PTCy‐based transplantation [12]. Despite continuous advances in donor selection, conditioning strategies, and GvHD prophylaxis, relapse remains a major cause of treatment failure after alloHSCT. Therefore, current efforts are focused on optimizing the balance between GvL activity and GvHD while minimizing transplant‐related toxicity.

Dissecting immunological processes in the postallotransplantation environment has brought to the forefront mechanisms of immune escape, including loss of mismatched human leukocyte antigen (HLA) and downregulation of HLA molecules in leukemia cells, dampening immune response with anti‐inflammatory cytokines, and overexpansion of the immune suppressor cell population [9]. The latter has been widely described in the context of regulatory T cells (Tregs) and their expression of immune checkpoints (ICPs) [13]. Yet, some populations of monocytic cells have attracted interest for restoring the balance between the GvL effect and GvHD, given their role as immunoregulators and suppressors of alloreactive T cells [14].

While donor‐derived alloreactive immune cells are essential for the eradication of residual malignant cells, excessive immune activation may lead to severe tissue damage and transplant‐related morbidity. Growing evidence suggests that selected myeloid populations may contribute to regulating these opposing processes by modulating T‐cell responses and inflammatory signaling. In this review, we summarize current knowledge on the roles of nonclassical monocytes, M2‐polarized macrophages, and myeloid‐derived suppressor cells (MDSCs) in the postallotransplantation environment, with particular emphasis on their potential to balance GvHD and GvL effects, thereby supporting immune tolerance while preserving antitumor immunity. To provide the necessary biological and clinical context, we first outline the key mechanisms underlying GvHD and GvL responses before discussing the contribution of these immunoregulatory myeloid populations in the posttransplant setting.

## 2. GvHD Overview

Despite decades of extensive research, the immunopathogenesis of GvHD remains only partially elucidated. Contemporary models support a multiphase process initiated by conditioning‐induced tissue damage, primarily caused by chemotherapy and total body irradiation. This initial injury facilitates the release of danger‐associated molecular patterns (DAMPs), proinflammatory cytokines—including tumor necrosis factor‐alpha (TNF‐α), interleukin‐1 (IL‐1), and interleukin‐6 (IL‐6)—and chemokines such as CCL2–5 and CXCL9–11. Consequently, there is an upregulation of adhesion molecules, major histocompatibility complex (MHC) antigens, and costimulatory signals on recipient antigen‐presenting cells (APCs) [15]. Injuries related to conditioning affecting the gastrointestinal tract assume a pivotal role by facilitating the systemic translocation of microbial products such as lipopolysaccharide (LPS), thereby further enabling host APC activation and exacerbating inflammatory cascades [16]. The extent of donor–recipient HLA disparity, as observed in haploidentical transplantation, broadens the alloantigen repertoire and elevates the risk of GvHD [17]. aGvHD is initiated when alloreactive donor T cells recognize recipient alloantigens, which are primarily presented by conditioning‐resistant recipient APCs, leading to donor T‐cell priming, clonal expansion, and differentiation of donor T cells into cytotoxic effector cells that target conventional GvHD organs, such as the skin, liver, lungs, and gastrointestinal tract [17]. Experimental studies have demonstrated that recipient nonhematopoietic APCs within target tissues are sufficient to induce lethal aGvHD, underscoring their critical role in disease initiation and propagation [18]. The effector phase of aGvHD is characterized by extensive tissue injury mediated by cytolytic pathways and sustained cytokine release from both adaptive and innate immune cells, culminating in epithelial apoptosis and organ dysfunction [17].

Historically, cGvHD was defined primarily by its temporal onset beyond 100 days after transplantation; however, contemporary classification emphasizes distinct clinical and pathobiological features, most notably progressive end‐organ dysfunction and fibrosis [19]. Similar to aGvHD, the early phase of cGvHD is initiated by tissue injury and inflammatory cytokine release, but disease evolution is subsequently shaped by persistent immune dysregulation rather than acute inflammation [20]. Thymic damage and impaired central tolerance promote aberrant B‐ and T‐cell reconstitution, characterized by accumulation of Th17/Tc17 and T follicular helper (Tfh) cells and a concomitant reduction in Treg function [20, 21]. In a second phase, sustained production of profibrotic and immunomodulatory cytokines, including transforming growth factor (TGF)‐β, IL‐17 derived from Th17 cells, and IL‐21 produced by Tfh cells, drives pathological antibody production and perpetuates immune activation [21, 22]. The final phase of cGvHD is marked by defective tissue repair and chronic inflammation, ultimately resulting in fibrotic remodeling of target organs [9, 20, 22]. Thus, in contrast to the predominantly cytokine‐driven, T‐cell–mediated inflammatory pathology of aGvHD, cGvHD displays autoimmune‐ and alloimmune‐like features, arising from dysregulated and persistent interactions among T cells, B cells, macrophages, dendritic cells (DCs), and neutrophils, which converge on profibrotic pathways and lead to progressive tissue scarring and irreversible organ damage.

## 3. The Relapse After Allotransplantation at Glance

The therapeutic effect of alloHSCT largely relies on the GvL phenomenon, mediated by transferred immune cells, primarily T cells and natural killer (NK) cells. However, relapse occurs in ~40% of patients and remains the leading cause of mortality post‐alloHSCT [6, 23]. One of the most well‐described immune evasion mechanisms in AML blasts is the impaired recognition by NK or T cells, resulting from disrupted leukemia cell recognition due to either nongenomic aberrations in HLA expression or HLA loss [24].

Moreover, HLA downregulation often occurs independently of other immune escape mechanisms, such as the dysregulation of inhibitory ICPs. In healthy individuals, ICPs on immune effector cells serve as crucial regulatory mechanisms to maintain immune tolerance and prevent autoimmunity. However, cancer cells can exploit these pathways to evade antitumor immune responses by expressing ligands for ICPs. The PD‐1/PD‐L1 axis is a major inhibitory pathway where PD‐1, expressed on various immune cells including T‐cell subtypes, binds to its ligand PD‐L1 on AML blasts. Through the PD‐1/PD‐L1 interaction, leukemic cells can induce T‐cell exhaustion and recruit Tregs, leading to T cell suppression, which has been associated with AML relapse after alloHSCT [13].

While PD‐1/PD‐L1 signaling is one of the best‐characterized pathways, the overexpression or upregulation of other checkpoints on T cells or corresponding ligands on AML blasts has also been reported, including CTLA‐4, TIM‐3, KLRG‐1, CD47, CD200, and LAG3 [25]. This highlights the complexity of immune pathways that impede effective antitumor responses. Additionally, the involvement of immunosuppressive cell subsets such as Tregs, MDSCs, and leukemia‐associated macrophages (LAMs) in remodeling the tumor microenvironment (TME) further contributes to immune evasion and diminishes the GvL effect [26].

In summary, the dysfunction of immune effector cells contributes to tumor immune escape by remodeling the TME towards an immunosuppressive phenotype (Table 1).

**Table 1:** The summary of the described effects of the monocyte and macrophage subpopulations on the postallotransplantation environment.

Subpopulation	GvL effect	Acute GvHD effect	Chronic GvHD effect	Literature
Classical monocytes	↑	↓	↓	Choi et al. [15]
Intermediate monocytes	↓	↑	↑	Koyama et al. [16]
Nonclassical monocytes	↓	↑	↑	Demosthenous et al. [14]
Leukemia‐associated macrophages (LAM)	↓	—	↑	Lee [19], Shlomchik [17]
M1‐type macrophages	↑	↑	↓	Zeiser and Vago [25], Lee [19]
M2‐type macrophages	↓	↓	↑	Bashey et al. [12], Turcotte et al. [27], Reinhardt et al. [28]
Monocytic myeloid–derived suppressor cells (M‐MDSCs)	↓	↓	↓	Srivastava et al. [29], Tang et al. [30]
Immature polymorphonuclear myeloid–derived suppressor cells (PMN‐MDSCs)	↓	↓	↓	Mantovani et al. [31], Tang et al. [30]

## 4. Monocytes

Monocytes are a heterogeneous group of cells divided into three subpopulations: classical (CD14^+^CD16^−^), intermediate (CD14^+^CD16^+^), and nonclassical (CD14^+^CD16^++^). Classical monocytes, known for their phagocytic activity and the production of anti‐inflammatory cytokines like IL‐10, are involved in tissue repair and innate immunity. Nonclassical monocytes, on the other hand, are more inflammatory, producing cytokines such as TNF‐α and IL‐1β, and play a key role in immune surveillance [32]. Intermediate monocytes exhibit characteristics of both classical and nonclassical types and are expanded during acute inflammation. Research indicates that recovery of classical monocytes is crucial for positive transplant outcomes, with delayed recovery linked to poorer survival rates and increased relapse risk [27]. This underscores the importance of monocyte subpopulations in immune recovery, likely due to their roles in cytokine production and antigen presentation.

**Figure 1:**
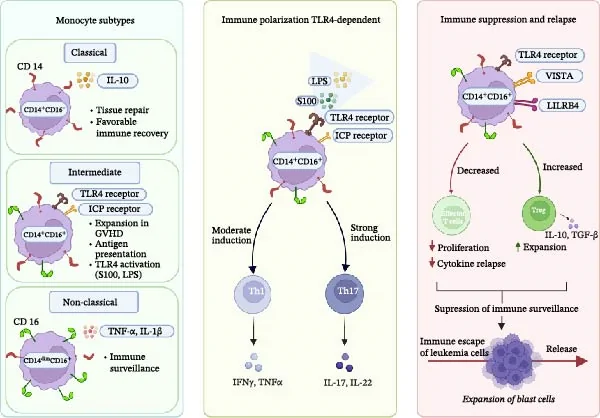
Monocyte subset–dependent regulation of GVHD and immune escape after alloHSCT. Intermediate CD14^+^CD16^+^ monocytes expand during GVHD and promote Th17‐skewed alloimmune responses via TLR4‐dependent inflammatory signaling, while immune checkpoint expression (VISTA, LILRB4) suppresses antileukemia T‐cell activity and enhances regulatory T‐cell expansion, contributing to immune escape after alloHSCT.

Intermediate monocytes isolated from patients with acute or cGvHD significantly increase Th17 cell levels in vitro compared to those without GvHD or healthy donors, suggesting that activated monocytes play a pivotal role in GvHD development [28]. These monocytes also induce higher Th1 cell levels, though the increase is less pronounced than Th17 cells [28]. Studies by Reinhardt et al. indicate that proinflammatory S100 proteins can enhance Th17 cell induction by binding to TLR4 on monocytes, aligning with earlier findings that showed further enhancement of Th17 levels when monocytes were stimulated with the TLR4 ligand LPS [28]. Eventually, monocytes express on their surface ICPs, such as the V‐domain Ig suppressor of T cells (VISTA) and LILRB4. T‐cell antileukemia responses are inhibited through the ICP activation, which reduces T‐cell proliferation and cytokine production while enhancing Treg activity, primarily via the VISTA signaling pathway [33]. Pagliuca et al. revealed that VISTA expression levels correlate with AML recurrence after chemotherapy treatment within 2 years from diagnosis [34]. VISTA and LILRB4 blockades may be considered attractive strategies for fine‐tuning leukemia immune control; however, their role regarding relapse after alloHSCT remains unclear. The summary of monocytes’ participation in GvHD and relapse mechanisms is illustrated in Figure 1.

## 5. Macrophages

Macrophages are characterized by substantial heterogeneity and functional plasticity. In the context of AML, recipient‐derived macrophages, commonly referred to as LAMs, display predominantly anti‐inflammatory and immunoregulatory properties and contribute to leukemic immune escape [31]. These host macrophages exhibit increased expression of multiple ICP molecules, including TIGIT, TIM‐3, and LAG‐3, as well as VISTA and LILRB4, as discussed in the previous section [18]. In addition, AML blasts can secrete arginase II, which drives the polarization of recipient macrophages from a proinflammatory M1 phenotype toward an immunosuppressive M2‐like state, thereby further promoting immune evasion [35].

In GvHD, host macrophages that survive conditioning regimens and persist after transplantation play a central immunomodulatory role through phenotypic polarization, cytokine production, and crosstalk with donor‐derived immune cells, particularly T lymphocytes. Macrophage polarization reflects the acquisition of distinct functional programs in response to environmental cues and gives rise to two broadly defined subsets: classically activated proinflammatory M1 macrophages and alternatively activated anti‐inflammatory M2 macrophages. These divergent activation pathways are tightly regulated by arginine metabolism. While the inducible nitric oxide synthase (iNOS) pathway, converting arginine into citrulline and nitric oxide (NO), supports M1‐like macrophage differentiation, M2 polarization is promoted by the arginase pathway, which converts arginine into ornithine and urea [36].

In the setting of acute tissue injury or inflammation, recipient macrophages initially adopt an M1 phenotype and produce proinflammatory cytokines such as TNF‐α, IL‐1β, IL‐12, and IL‐23 to limit tissue damage and control immune activation. However, sustained M1 activity may exacerbate tissue injury. To counterbalance this response, polarization toward an M2 phenotype occurs, with host macrophages secreting high levels of IL‐10 and TGF‐β, thereby suppressing inflammation, promoting tissue repair and remodeling, supporting vasculogenesis, and facilitating the restoration of tissue homeostasis [31, 36].

Macrophage infiltration and phenotype are closely linked to GvHD severity and disease stage. In aGvHD, recipient macrophages predominantly exhibit an M1‐like, proinflammatory profile, whereas in steroid‐refractory aGvHD and cGvHD, M2 polarization becomes increasingly prevalent [37]. Through cytokine secretion, antigen presentation, and expression of ICP molecules, host macrophages directly regulate donor T‐cell activation and differentiation [36]. Notably, macrophages can promote immune tolerance by dampening effector T‐cell responses and facilitating Treg differentiation via checkpoint‐mediated signaling pathways [38].

The expansion of Tregs further strengthens this regulatory circuit by suppressing M1 polarization and restricting effector T‐cell infiltration, especially in the presence of IL‐33 during aGvHD [39]. Functionally, M1 macrophages predominantly facilitate the priming of CD8^+^ T cells, whereas M2 macrophages inhibit CD8^+^ T‐cell infiltration and activity, thereby supporting immune homeostasis [40].

Importantly, host macrophages differ fundamentally from donor‐derived macrophages in their interaction with donor T cells. Seminal research by Hashimoto et al. demonstrated that depletion of recipient macrophages enhances donor T‐cell expansion and exacerbates GvHD‐associated mortality through a CD47‐dependent mechanism [41]. Conversely, the persistence of host macrophages following transplantation mitigates GvHD by engulfing donor alloreactive T cells and inhibiting their proliferation. In cases of cGvHD, intricate interactions among CD4^+^ T cells, fibroblasts, B cells, and recipient macrophages promote tissue remodeling and fibrosis. M2‐polarized macrophages play a crucial role in activating fibroblasts and advancing fibrosis, predominantly mediated by IL‐4 and TGF‐β1 [42]. Notably, the M2c macrophage subset may exert antifibrotic effects, particularly within pulmonary tissues, by increasing IL‐10 production [30]. Furthermore, regulatory macrophage populations have the capacity to inhibit alternative macrophage activation and limit fibrosis induced by profibrotic macrophage subsets [43].

**Figure 2:**
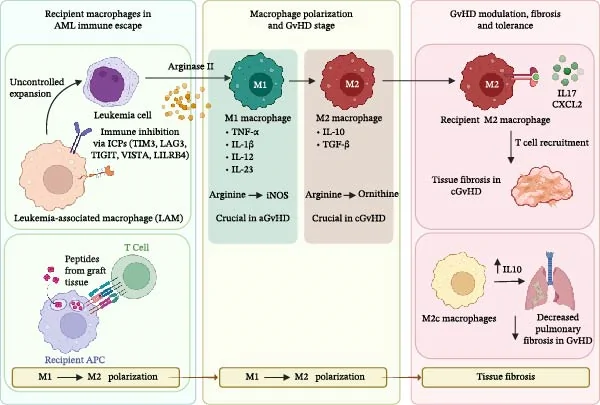
Recipient‐derived macrophages as central regulators of GVHD severity, immune tolerance, and fibrosis after alloHSCT. Recipient‐derived macrophages persist after alloHSCT and act as central regulators of immune activation, tolerance, and tissue remodeling. Through dynamic polarization between proinflammatory M1 and immunosuppressive M2 phenotypes, metabolic reprogramming of arginine utilization, and expression of immune checkpoint molecules, host macrophages shape GVHD severity, promote immune tolerance, and contribute to fibrotic complications characteristic of chronic GVHD.

Cytokines are key regulators of macrophage performance in GvHD. For instance, CXCL2, also known as macrophage inflammatory protein, is crucial in recruiting macrophages and T cells to target organs in GvHD [44]. Additionally, IL‐17 produced by Th17 cells participates in GvHD by modulating interactions between macrophages and CD4^+^ T cells, reducing macrophage infiltration, downregulating IL‐12 and IFN‐γ production, suppressing Th1 responses, and ultimately alleviating aGvHD [45]. The role of macrophages in the subsequent stages of GvHD is illustrated in Figure 2.

## 6. MDSCs

MDSCs are widely described as myeloid cells, including myeloid progenitors and immature mononuclear cells. Based on their morphological and phenotypic similarity, they are divided into the following subsets: monocytes, MDSCs (M‐MDSCs), and immature polymorphonuclear MDSCs (PMN‐MDSCs) [46]. The primary function of MDSCs is their suppressive activity against other immune cells, mainly by suppressing effector T‐cell responses and promoting the Tregs development [47]. MDSCs can mediate immune suppression through multiple mechanisms: producing reactive oxygen species [48] and inducible NOS [49], depleting key amino acids required for T cell proliferation[29], and producing immunosuppressive cytokines such as TGF‐β and IL‐10 [50, 51]. Moreover, MDSCs can inhibit NK cell cytokine production [52]. MDSCs also express high levels of arginase, which transports extracellular arginine and consumes it, leading to insufficient arginine for T cells and, therefore, impaired T‐cell–mediated antitumor immunity [53]. M‐MDSCs evade both antigen‐specific and nonspecific T‐cell responses and exhibit more potent activity than PMN‐MDSCs, which can only suppress immune responses in an antigen‐specific manner [54]. Eventually, MDSCs exert immunosuppressive activity via ICP signaling pathways by expressing VISTA and PD‐L1, a ligand for the PD‐1 receptor [55]. MDSCs have been described in peripheral blood, lymphoid organs, spleen, and tumor sites in the settings of cancer, infection, chronic inflammation, and, more recently, transplantation and autoimmunity [46, 51]. Although MDSCs are barely detectable in the peripheral blood of healthy individuals, their numbers increase in cancer settings, where they promote immune evasion [56].

The role of MDSCs in tumor progression, including leukemia and MDS, has been broadly described. Yet, their participation in post‐alloHSCT immunologic balance has been reported ambiguously. MDCS population is prevalent during early hematopoietic recovery and continues to exert a T‐cell suppressive effect up to 3 weeks post‐alloHSCT [57]. Furthermore, regarding their suppressive activity and promoting Treg development, their high level in the early period after alloHSCT has been correlated with a higher risk of relapse after alloHSCT [26]. This finding contrasts with the research of Gournay et al., who showed that, unlike the consistently high Treg levels, MDSC subsets increase soon after transplantation and then progressively decrease over time. Thus, no correlation was found between MDSC levels and subsequent relapse [58]. This statement is further substantiated by findings from murine models, which show that MDSCs not only alleviate GvHD‐associated inflammation but also preserve the GvL effect [51, 59]. Furthermore, within the first 100 days post‐alloHSCT, elevated MDSC levels in peripheral blood were associated with the development of severe aGvHD, without a corresponding increase in the risk of malignancy relapse [60]. Notably, studies that reported increased MDSC frequencies also observed a corresponding rise in Treg levels. This link between MDSCs and Tregs has been demonstrated in both murine models and clinical research. Moreover, studies involving MDSC depletion showed a subsequent reduction in Treg populations, underscoring the critical role of MDSCs in enhancing Treg activity to mitigate GvHD [59].

Donor lymphocyte infusion (DLI), administered to establish full donor chimerism or to prevent and treat relapse after alloHSCT, has been evaluated both with and without G‐CSF pretreatment in donors. PBSC‐derived DLIs collected post‐G‐CSF treatment promoted the transition to full donor chimerism, despite a higher presence of M‐MDSCs [51]. Additionally, DLIs treated with G‐CSF were linked to reduced rates of relapse and disease progression, without significantly increasing the overall incidence of GvHD [61]. Rieber et al. identified significantly higher levels of MDSCs in the peripheral blood of patients undergoing extracorporeal photopheresis (ECP) for GvHD treatment than in both healthy controls and alloHSCT patients without GvHD [62].

Primary targets of aGvHD are the skin, liver, and intestines. However, data linking GvHD manifestations to MDSCs are limited. Notably, a clinical study by Vendramin et al. found that G‐CSF induced M‐MDSCs, which mitigated aGvHD by reducing tissue damage and inflammation across all affected organs [63]. Additional studies in xenogeneic GvHD models have supported these findings. For instance, D’Aveni et al. reported reduced GvHD histopathological scores, especially in the colon, following the infusion of G‐CSF–mobilized CD34^+^ monocytes [64]. Similarly, Wang et al. observed improved histopathological scores in liver and intestine tissues when G‐CSF mobilized HLA‐DR^−^/lowCD33^+^CD16^−^ cells were administered [65].

**Figure 3:**
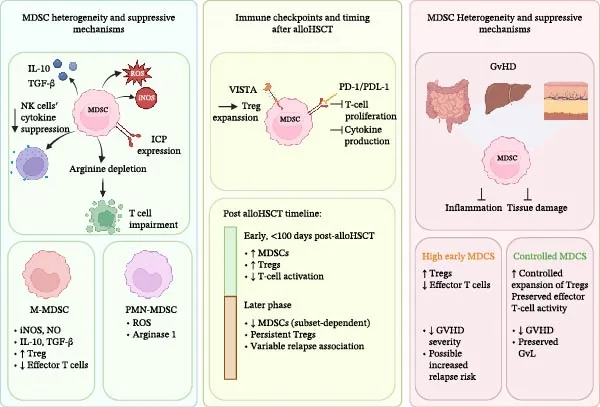
Myeloid‐derived suppressor cells as regulators of immune suppression, GVHD severity, and GvL after alloHSCT. MDSCs, including M‐MDSCs and PMN‐MDSCs subsets, expand in inflammatory and malignant settings and exert potent immunosuppressive functions. Through production of reactive oxygen species, inducible nitric oxide synthase, arginase‐mediated arginine depletion, and immunosuppressive cytokines such as IL‐10 and TGF‐β, MDSCs suppress effector T‐cell and NK‐cell responses while promoting Treg expansion. MDSCs also express immune checkpoint molecules, including VISTA and PD‐L1, further dampening antileukemic immunity. After alloHSCT, MDSCs transiently expand during early hematopoietic recovery, mitigating GVHD‐associated tissue inflammation while exerting context‐ and timing‐dependent effects on graft‐versus‐leukemia activity.

Findings from both murine models and clinical studies indicate that MDSCs play an important immunoregulatory role in the post‐alloHSCT environment. By suppressing alloreactive T‐cell responses and promoting Treg expansion, these cells may attenuate GvHD‐associated tissue damage while potentially preserving GvL activity. The described mechanisms are illustrated in Figure 3. Although the exact influence of MDSCs on the risk of relapse and long‐term immune surveillance is not fully understood, growing evidence indicates that their functional characteristics may hold therapeutic significance. These observations have therefore stimulated growing interest in MDSC‐based strategies to modulate posttransplant immune responses, particularly for the prevention and treatment of GvHD.

## 7. Therapeutic Perspectives in GvHD and GvL Modulation

Recent advances in understanding monocyte heterogeneity and myeloid cell plasticity after alloHSCT have provided a strong rationale for developing therapeutic strategies to selectively modulate GvHD while preserving GvL activity. Recent approaches leverage myeloid checkpoint modulation using agents such as LILRB4 and PD‐L1, as well as engineered suppressive myeloid cells and optimized regimens of PTCy and bendamustine to reprogram posttransplant immune responses [62, 63, 65–70]. These interventions are supported by recent preclinical and early clinical data that demonstrate the possibility of targeted immunoregulation without wholesale myeloablation. The subsequent overview highlights significant recent advances, with particular focus on the most promising translational pathways.

G‐CSF, ECP,PTCy, and bendamustine have all been shown to promote the expansion or functional reprogramming of immunoregulatory monocytes, macrophages, and MDSCs, thereby contributing to immune tolerance and mitigation of GvHD [62, 64, 68, 69]. ECP induces immunoregulatory changes in monocytes that suppress alloreactive T‐cell proliferation through contact‐dependent mechanisms involving PD‐L1/PD‐1 signaling, thereby promoting peripheral tolerance [71]. In parallel, ECP has been shown to expand suppressive PMN‐MDSCs that attenuate Th1 and Th17 responses through arginase‐dependent pathways, providing an additional mechanism to control GvHD‐associated inflammation [62]. These findings support a central role for ECP‐mediated myeloid reprogramming in establishing immune tolerance after alloHSCT while potentially preserving beneficial GvL activity.

PTCy is an established standard for GvHD prophylaxis that indirectly leverages myeloid regulation. In addition to eliminating alloreactive T cells, high‐dose PTCy induces a wave of regulatory myelopoiesis. In both murine models and human patients, PTCy significantly expands monocytic and granulocytic MDSCs during the early posttransplant period [72]. In humans, compared to antithymocyte globulin, PTCy‐treated patients demonstrate higher circulating CD14^+^HLA‐DR^low^ MDSCs and CD4^+^FoxP3^+^ Tregs in the initial weeks following HSCT, indicative of accelerated myeloid–regulatory recovery [73]. Recent clinical data further support the role of MDSCs in PTCy‐mediated immune tolerance. In a prospective comparison of PTCy and standard GvHD prophylaxis, PTCy recipients exhibited enhanced early recovery of functional PMN‐MDSCs, which retained potent T‐cell suppressive activity and were associated with lower rates of both acGvHD and cGvHD, highlighting MDSCs as key mediators of posttransplant immune regulation [69, 74]. Timing is of critical importance: The administration of PTCy on days +3–4 effectively eradicates proliferating alloreactive T cells, and the subsequent bone marrow environment—predominantly influenced by homeostatic cytokines—favors the development of suppressive myeloid cells [69].

Bendamustine, a unique alkylating agent with purine analog properties, has emerged as a promising alternative to PTCy for GvHD prophylaxis. In murine alloHSCT models, both pre‐ and posttransplant bendamustine reduced GvHD while preserving or enhancing GvL activity, effects associated with the expansion and functional enhancement of M‐MDSCs in the blood, bone marrow, spleen, and GvHD target tissues [75]. Bendamustine directly augments the suppressive capacity of MDSCs against activated T cells and promotes an arginase‐1–dependent regulatory program, resulting in L‐arginine depletion and inhibition of alloreactive T‐cell proliferation and IFN‐γ production, without compromising antileukemic immunity [68]. Compared with PTCy, bendamustine has demonstrated equivalent GvHD control with superior GvL preservation, reduced myelosuppression, faster hematopoietic recovery, and enhanced MDSC expansion, supporting its further development as a myeloid‐directed immunomodulatory strategy after alloHSCT [51, 68].

Findings by Zhang et al. indicate that myeloid‐cell–mediated immune regulation is highly context‐ and time‐dependent, as MDSCs suppress early alloreactive responses and GvHD while preserving later antileukemic immunity, highlighting the importance of precise therapeutic modulation rather than broad myeloid suppression [58].

Similar protection has been achieved using pegylated recombinant arginase‐1, highlighting this metabolic pathway as a potential noncellular therapeutic target.

The foundational demonstration of this principle came from work showing that M‐MDSCs generated from the bone marrow of nontumor‐bearing donors through short‐term culture with G‐CSF and GM‐CSF potently suppress allogeneic T‐cell responses and reduce GvHD lethality through an arginase‐1–dependent depletion of L‐arginine, which limits donor T‐cell proliferation, activation, and IFN‐γ production. The addition of IL‐13 to these cultures further upregulates arginase‐1 and yields a more suppressive monocytic subset (MDSC–IL‐13) that, critically, attenuates GvHD while leaving the GvL activity of donor T cells intact in a lymphoma challenge model [76]. The protective effect was largely reproduced by in vivo administration of a pegylated form of arginase‐1, reinforcing L‐arginine catabolism as a tractable, monocyte‐associated mechanism for uncoupling alloreactive tissue injury from antileukemic immunity [76].

Translation of MDSC‐based therapy is nonetheless constrained by the instability of the suppressive phenotype within the inflammatory milieu of aGvHD. Adoptively transferred MDSCs are exposed to conditioning‐ and alloreactivity‐derived danger signals that drive their conversion to a mature, inflammasome‐activated state, marked by caspase‐1 processing and IL‐1β secretion, with a concomitant loss of arginase‐1 expression and suppressive function [77]. This inflammasome‐mediated loss of function can be addressed genetically: Donor MDSCs deficient in the inflammasome adaptor ASC (encoded by Pycard) retain suppressive capacity and confer superior GvHD protection relative to wild‐type cells, while repeated MDSC infusions during the early posttransplant window can compensate for the progressive functional exhaustion of previously transferred cells [77]. These observations capture both the promise and the central challenge of myeloid cell therapy: The same plasticity that permits rapid in vitro generation of highly suppressive cells also renders them susceptible to reprogramming by the GvHD microenvironment, such that strategies stabilizing the regulatory phenotype—through genetic engineering, selective inflammasome inhibition, or optimized dosing—will be required to realize its full therapeutic potential.

Emerging pharmacological strategies increasingly focus on pathways central to myeloid‐mediated immune regulation, such as ICP pathways. Targeting these pathways may rebalance monocyte and macrophage polarization and restore effector T‐cell function without fully abrogating immune tolerance [35, 36]. In parallel, ICP molecules expressed by myeloid cells, such as VISTA, LILRB4, and PD‐L1, have gained attention as potential therapeutic targets [78–80].

VISTA (also known as PD‐1H) agonists have demonstrated efficacy in preclinical studies. The research by Koehn et al. demonstrated that donor T cells are central mediators of GvHD and that VISTA‐targeting antibody treatment significantly reduced the accumulation and infiltration of alloreactive donor T cells in GvHD target organs through Fc receptor–dependent phagocytic deletion, thereby resulting in reduced T‐cell infiltration of target tissues and mitigating disease severity [79]. Agonistic antibodies targeting VISTA (expressed on phagocytic cells such as neutrophils and macrophages) therefore offer potential for inducing immune tolerance without compromising GvL effects [79]. Another inhibitory receptor, LILRB4 (ILT3), is predominantly expressed on monocytic leukemias and immunosuppressive myeloid cells. Recent research has identified LILRB4 as a marker of monocytic AML that mediates the suppression of T‐cell activity [81]. This discovery has led to preclinical therapeutic approaches, such as “STAR‐T” cells (CAR‐T cells engineered to target LILRB4), which effectively eliminate LILRB4‐positive AML while preserving normal hematopoiesis [82]. Although primarily focused on antileukemia strategies, these approaches highlight the potential of the LILRB4 blockade to reverse myeloid‐mediated immune tolerance. Conversely, broad blockade of PD‐1/PD‐L1 following alloHSCT is highly effective in reactivating antitumor immunity but is associated with a significant risk of GvHD. Studies of post‐alloHSCT lymphoma relapse indicate that PD‐1 inhibitors (e.g., nivolumab and pembrolizumab) can trigger or worsen GvHD even when administered with steroid coverage [83]. Consequently, current research has increasingly focused on selective modulation of the PD‐1/PD‐L1 axis and other context‐dependent immune pathways to enhance GvL activity while minimizing GvHD, rather than relying on broad immune activation [84].

The most direct clinical validation of myeloid‐targeted therapy has been derived from CSF‐1R blockade in cGvHD. Monocyte‐derived macrophages, particularly CSF‐1/CSF‐1R–dependent profibrotic M2 macrophages, are now acknowledged as pivotal effectors of tissue inflammation and fibrosis characteristic of chronic diseases. In murine models of sclerodermatous and pulmonary cGvHD, the disease develops in a manner dependent on CSF‐1/CSF‐1R and is significantly mitigated by antibody‐mediated depletion of donor‐derived macrophages [85]. Axatilimab, a humanized IgG4 monoclonal antibody that inhibits ligand binding to CSF‐1R, exploits this dependency by depleting and reprogramming pathogenic monocytes and macrophages. In the critical Phase 2 AGAVE‐201 trial, axatilimab achieved overall response rates ranging from 50% to 74% across three dose levels in patients with recurrent or refractory cGvHD who had undergone a median of four prior treatment lines, with responses that were durable across all affected organs, including fibrotic manifestations of the skin, lungs, and fascia. This led to FDA approval in 2024 [85, 86]. Notably, the therapeutic window is dependent on timing in a manner that parallels the experimental MDSC data: whereas early peritransplant CSF‐1R blockade may exacerbate aGvHD by depleting protective host macrophages, blockade during established cGvHD proves beneficial because the resident macrophage population has then been replaced by pathogenic donor‐derived cells [85, 86]. This temporal dichotomy underscores a recurring principle that interventions targeting monocytes and macrophages must be tailored to the disease phase and the prevailing inflammatory context, rather than being uniformly applied as myeloid suppression.

## 8. Conclusions

The monocytic landscape after alloHSCT remains complex but holds promise for further research, with potential targets to alleviate GvHD without compromising GvL, thereby improving allotransplant outcomes. However, there are still unmet needs in understanding the mechanisms that initiate and sustain GvL responses that are potentially distinct from those driving GvHD. A deeper understanding of these mechanisms could enhance the safety and accessibility of allogeneic transplantation, ultimately improving both survival and quality of life for patients.

## Author Contributions

**Magdalena Karasek:** Conceptualization, literature analysis, manuscript drafting, and revision. **Kinga Chmielewska:** Manuscript drafting, graphic preparation **Anna Czyż:** Manuscript review, editorial refinement, and critical revision for intellectual content.

## Funding

This study was supported by institutional funding from the Wroclaw Medical University (Grant SUBK.C140.25.035).

## Disclosure

All authors have read and approved the final version of the manuscript.

## Conflicts of Interest

The authors declare no conflicts of interest.

## Data Availability

Data sharing is not applicable to this article as no datasets were generated or analyzed during the current study.
